# Structural basis for dimerization of the death effector domain of the F122A mutant of Caspase-8

**DOI:** 10.1038/s41598-018-35153-5

**Published:** 2018-11-13

**Authors:** Chen Shen, Jianwen Pei, Xiaomin Guo, Lu Zhou, Qinkai Li, Junmin Quan

**Affiliations:** 1 State Key Laboratory of Chemical Oncogenomics, School of Chemical Biology and Biotechnology, Peking University Shenzhen Graduate School, Shenzhen, 518055 China; 2 School of Pharmacy, Fudan University, Shanghai, 201203 China

## Abstract

Caspase-8 is an apoptotic protease that is activated by a proximity-induced dimerization mechanism within the death-inducing signaling complex (DISC). The death effector domain (DED) of caspase-8 is involved in protein-protein interactions and is essential for the activation. Here, we report two crystal structures of the dimeric DEDs of the F122A mutant of caspase-8, both of which illustrate a novel domain-swapped dimerization, while differ in the relative orientation of the two subunits and the solvent exposure of the conserved hydrophobic patch Phe122/Leu123. We demonstrate that mutations disrupting the dimerization of the DEDs abrogate the formation of cellular death effector filaments (DEFs) and the induced apoptosis by overexpressed DEDs. Furthermore, such dimerization-disrupting mutations also impair the activation of the full-length caspase-8 and the downstream apoptosis cascade. The structures provide new insights into understanding the mechanism underlying the activation of procaspase-8 within the DISC and DEFs.

## Introduction

Caspase-8 is a cysteine protease that initiates the extrinsic apoptotic pathway in response to cell surface death receptor activation^1,2^. Procaspase-8 exists in the cytosol within the cell as an inactive monomer, which is characterized by an N-terminal tandem death effector domains (DEDs) and a C-terminal catalytic protease domain^3,4^. The activation of procaspase-8 is proposed to occur through an induced proximity mechanism^5–7^. Upon engagement with the death ligand such as Fas Ligand (FasL), the clustering death receptor Fas recruits the adaptor protein Fas-associated death domain (FADD) to its cytoplasmic tail^8^. The inactive procaspase-8 is then recruited to FADD via homotypic interactions between the DEDs of procaspase-8 and the adaptor protein FADD, leading to the dimerization and activation of procaspase-8^9^. During this process, dimeric FasL trimers potentially serve as a minimal unit fully capable of activating the Fas pathway^10^.

The current induced proximity model is proposed based on early studies using drug-induced dimerization of a truncated procaspase-8 without the DEDs^11,12^, or chimeric caspase-8 that Fpk3 or FKBP is fused to truncated procaspase-8 without the DEDs^5,9,13^. In these early studies, kosmotrope-induced dimerization of the caspase domain of procaspase-8 leads to its activation *in vitro*^12^. Moreover, the cell-permeable ligand-induced dimerization of the FKBP domain also drives the activation of the chimeric procaspase-8 in the intact cell^9^. However, the function of the DEDs in the activation of procaspase-8 cannot be fully addressed based on these studies using the truncated procaspase-8 that lacks the DEDs. It remains unclear whether the DEDs of procaspase-8 forms dimer, if true, how the DEDs forms the dimeric structure within the DISC.

To address these questions, we tried to determine the structure of the DEDs of caspase-8, but the structural study of the DEDs is markedly hindered by the low solubility and high aggregating propensity for the tandem DEDs of caspase-8^14^. Through mutant screening, we recently solved the structure of the monomeric DEDs of caspase-8 for a soluble F122A/I128D mutant (DED^F122A/I128D^)^15^, which closely resembles the structure of viral FLIP MC159^16,17^. However, the monomeric DED^F122A/I128D^ mutant provides limited clues about the homotypic interactions between the DEDs of caspase-8. More recently, the cryo-EM structure of caspase-8 tandem DED filaments was determined^18^, which provides an elegant structural framework to understand interactions between the DEDs of caspase-8 itself and that between caspase-8 and other proteins such as FADD, cFLIP, and vFLIP. This structure also takes a significant step forward in understanding the mechanism underlying death-inducing signaling complex (DISC) assembly. On the other hand, the filament formation generally occurs under high concentrations of DEDs *in vitro*^18^ or intracellularly^19–22^, it is unclear how the engagement of FasL triggers the downstream filament formation of caspase-8 under physiological condition. Moreover, further studies are still needed to address how the filament structure accommodate the requirement for dimerization in the activation of caspase-8, and how this structure reconciles with the observation that dimeric FasL trimer is a minimal unit fully capable of activating the Fas pathway^10^.

In this study, we determined the crystal structures of the DEDs of caspase-8 illustrating a novel domain-swapped dimer, which adopts either open or closed conformations in terms of the solvent-exposure of the conserved hydrophobic patch Phe122/Leu123 that is proposed to bind with the DED of FADD. Together with the biochemical data and the computational modeling, the domain-swapped structures of the dimeric DEDs of caspase-8 provide an alternative model to understand the dimerization-driven activation of caspase-8 in the Fas pathway mediated by a minimal dimeric FasL trimer.

## Results

### Soluble stable dimer of the DEDs mutant

**Figure 1:**
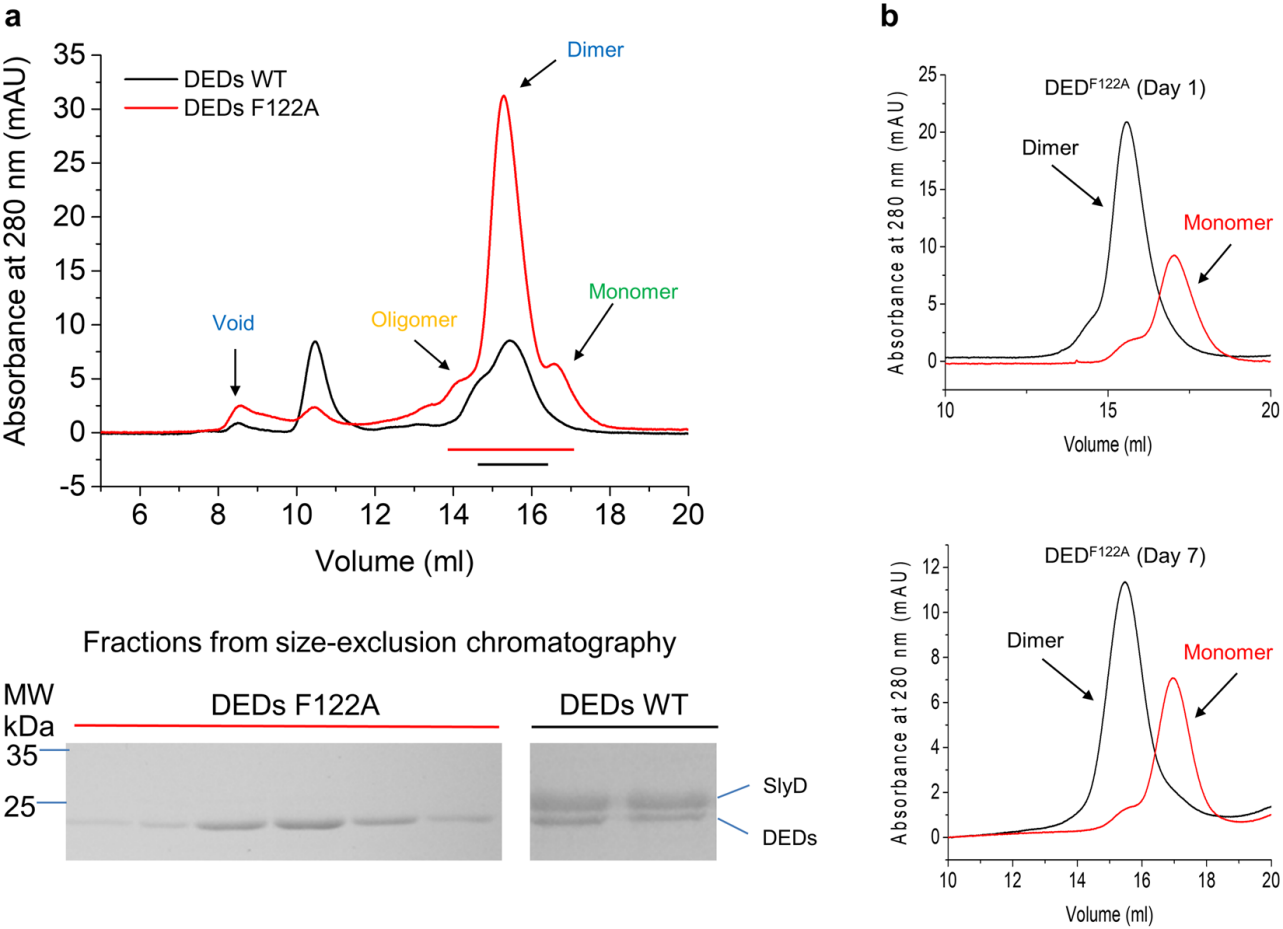
DEDs dimerization. (**a**) Gel filtration profiles of DED^WT^ and DED^F122A^ expressed without N-terminal fusion protein. The corresponding Coomassie stained SDS gel are shown at the bottom. (**b**) Gel filtration profile of the monomer and dimer of DED^F122A^ on the Superdex 200 10/300 GL column in Day 1 and 7, respectively. No obvious inter-conversion occurred during this period. The gels were cropped for clarity, and full-length gels were shown in Supplementary Fig. 9.

To eliminate the potential impact of the N-terminal fused proteins, such as GFP and MBP, on the homotypic interactions between the DEDs of caspase-8, we tried to express the wild-type DEDs with a C-terminal His6 tag. In consistent with the insoluble and aggregation-prone property of DEDs, we only obtained very low yields of soluble protein (Fig. 1a). Unexpectedly, the protein eluted from the peaks mainly corresponding to dimeric and oligomeric states rather than from the void position of the gel filtration column as shown in the previous study^18^, suggesting that dimeric DEDs rather than filamentous DEDs could be obtained under low expression level. However, further SDS-PAGE analysis showed that the DEDs dimer mixed with a bacterial folding helper protein slyD^23^, highlighting that the solubilization of the insoluble wild-type DEDs must be facilitated by slyD (Fig. 1a). In screening for the DED mutants that are soluble and remain self-assembled, we found a F122A mutant of the DEDs of caspase-8 (DED^F122A^), which is soluble and less aggregated (Fig. 1a). Interestingly, when expressed with an N-terminal His-SUMO fusion tag, the F122A mutant with tag removal still has dominant dimeric peak during gel filtration albeit having minor peaks corresponding to the monomeric and oligomeric states (Supplementary Fig. 1a). Both the DED^F122A^ monomer and dimer are highly stable at room temperature without obvious inter-conversion or aggregating in days (Fig. 1b). By contrast, both MBP-DED^WT^-SUMO and DED^Y8A^-SUMO form filaments in the previous study^18^, highlighting the different properties for DEDs under low and high expression levels.

### Crystal structure of dimeric DED^F122A^

**Figure 2:**
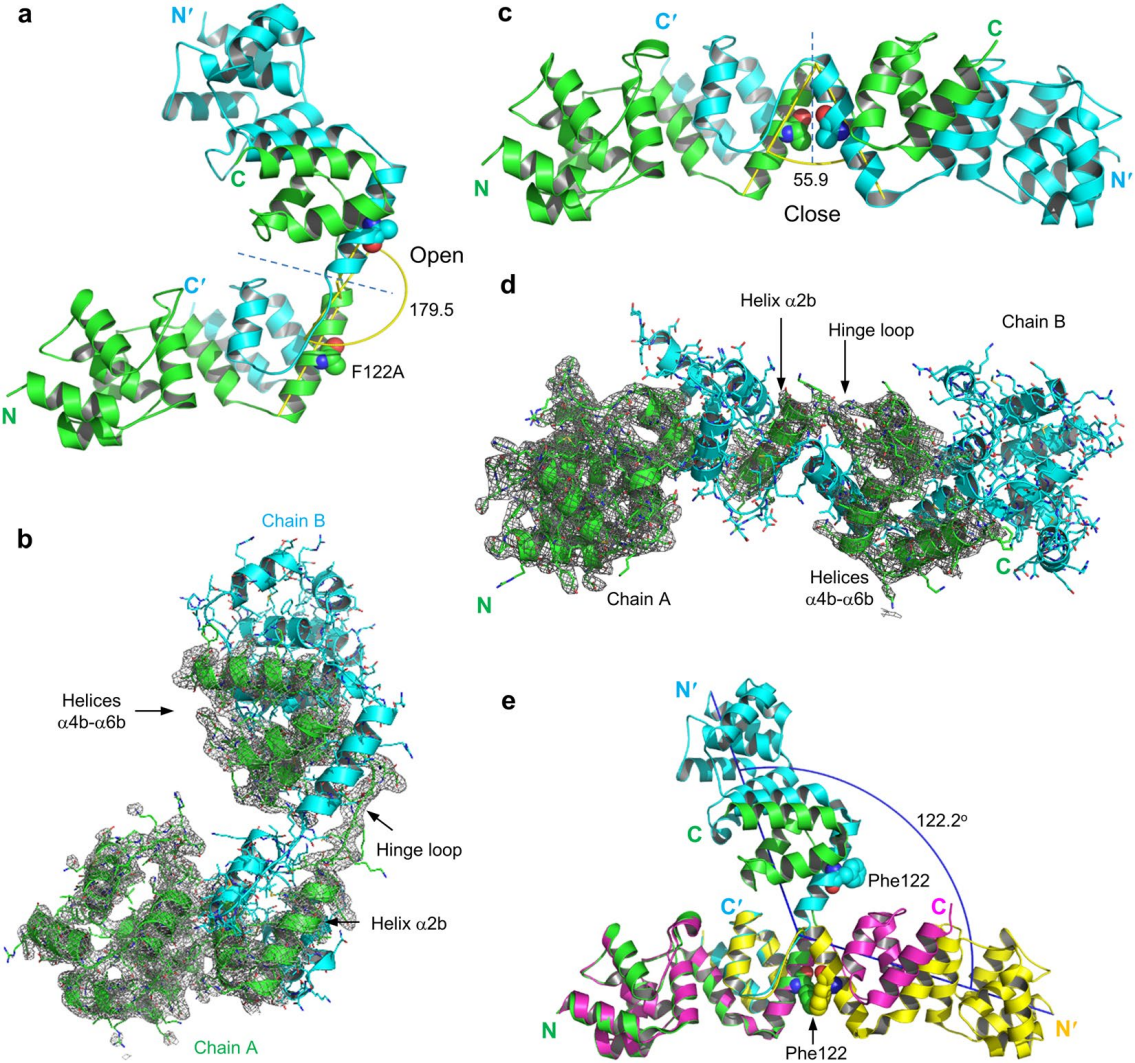
Structures of the dimeric DED^F122A^. (**a**) Structure of the domain-swapped dimeric DED^F122A^ in the open conformation, in which F122A is solvent-exposed. (**b**) 2Fo − Fc electron density map (contoured at 1.0σ) of chain A of the domain-swapped dimeric DED^F122A^ in the open conformation. (**c**) Structure of the domain-swapped dimeric DED^F122A^ in the closed conformation, in which F122A is buried at the interface of the dimer. (**d**) 2Fo − Fc electron density map (contoured at 1.0σ) of chain A of the domain-swapped dimeric DED^F122A^ in the closed conformation. (**e**) Structural superimposition of the domain-swapped dimeric DED^F122A^ in the open (PDB ID: 5H33, green and cyan) and closed (PDB ID: 5H31, magenta and yellow) conformations. Phe122 was modeled from F122A and highlighted by sphere representation. The orientation of globule modules was shown by the rotational angle (122.2 degrees).

To gain insight into homotypic interactions in dimeric DEDs, we set out to determine the crystal structure of the self-assembled DEDs. The dimeric peak was collected and further concentrated for crystallization (Supplementary Fig. 1a). We obtained crystals of two crystal forms with varied cell dimensions under different crystallization conditions. The crystal structures were determined to resolutions of 3.1 Å and 3.6 Å, respectively, in the *P*1 space group (Table 1). Both structures reveal a novel domain-swapped conformation involving the exchange of the C-terminal helices α4b-α6b between the two subunits, and the hinge loop for the domain swapping is located at the segment linking helices α2b and α4b (Fig. 2a–d). The overall structure of the dimer displays a dumbbell-like shape with two-fold molecular symmetry. Each globular module, containing helices α1a-α7a and α1b-α2b from one monomer and helices α4b-α6b from the other monomer (Supplementary Fig. 1b,c), closely resembles the crystal structure^15^ of the monomeric DED^F122A/I128D^ with a root mean squared deviation of 0.46 Å for 155 equivalent Cα atoms between them (Supplementary Fig. 1d). Such domain-swapped dimerization represents the fourth homotypic interaction type distinct from other three known interaction types in the death-fold superfamily^24^.

**Table 1:** Data collection and refinement statistics.

PBD ID	5H31	5H33
**Data collection**		
Wavelength (Å)	0.9779	1.5418
Space group	*P*1	*P*1
**Cell dimensions**		
*a*, *b*, *c* (Å)	51.77, 51.71, 171.96	56.37, 50.79, 90.31
*a*, *b*, *g* (°)	90.0, 90.0, 90.0	90.0, 105.4, 90.0
Resolution (Å)	50.0–3.15 (3.26–3.15)*	54.44–3.60 (3.73–3.60)*
Total reflections	88207	19517
*R*_merge_	0.166 (0.945)*	0.217 (0.560)*
*I*/s*I*	12.2 (2.0)*	4.4 (1.5)*
Completeness (%)	99.7 (99.6)*	99.6 (99.7)*
Redundancy	11.5 (22.6)*	3.31 (3.34)*
**Refinement**		
Resolution (Å)	49.59–3.17	43.63–3.60
Unique reflections	15236	5874
*R*_work_/*R*_free_	0.225/0.268	0.280/0.313
No. atoms	6064	3014
Protein	6064	3014
Ligand/Ion	—	—
Water	—	—
**B factors**		
Protein	73.0	86.0
Water		—
**r.m.s. deviations**		
Bond lengths (Å)	0.002	0.003
Bond angles (°)	0.475	0.644
**Ramachandran statistics**		
Most Favored (%)	96.25	96.37
Allowed (%)	3.75	3.63
Outlier (%)	0.0	0.0

*Values in parentheses are for highest-resolution shell.

The two dimeric crystal structures differ in the relative orientation of the two globule modules of the dimer, which represent the closed and open conformations in terms of the inter-helical angle between helices α2b from the monomeric subunits, or in terms of the solvent exposure of the conserved hydrophobic patch Phe122/Leu123 (Fig. 2e). The transition from the open conformation to the closed conformation buries about 500 Å^2^ solvent-accessible surface area, suggesting that the closed conformation might be more stable in solution. Whereas the open conformation with the solvent-exposed hydrophobic patch Phe122/Leu123 might facilitate the interaction with the partner proteins such as FADD in the DISC (Supplementary Fig. 2)^16,25^.

**Figure 3:**
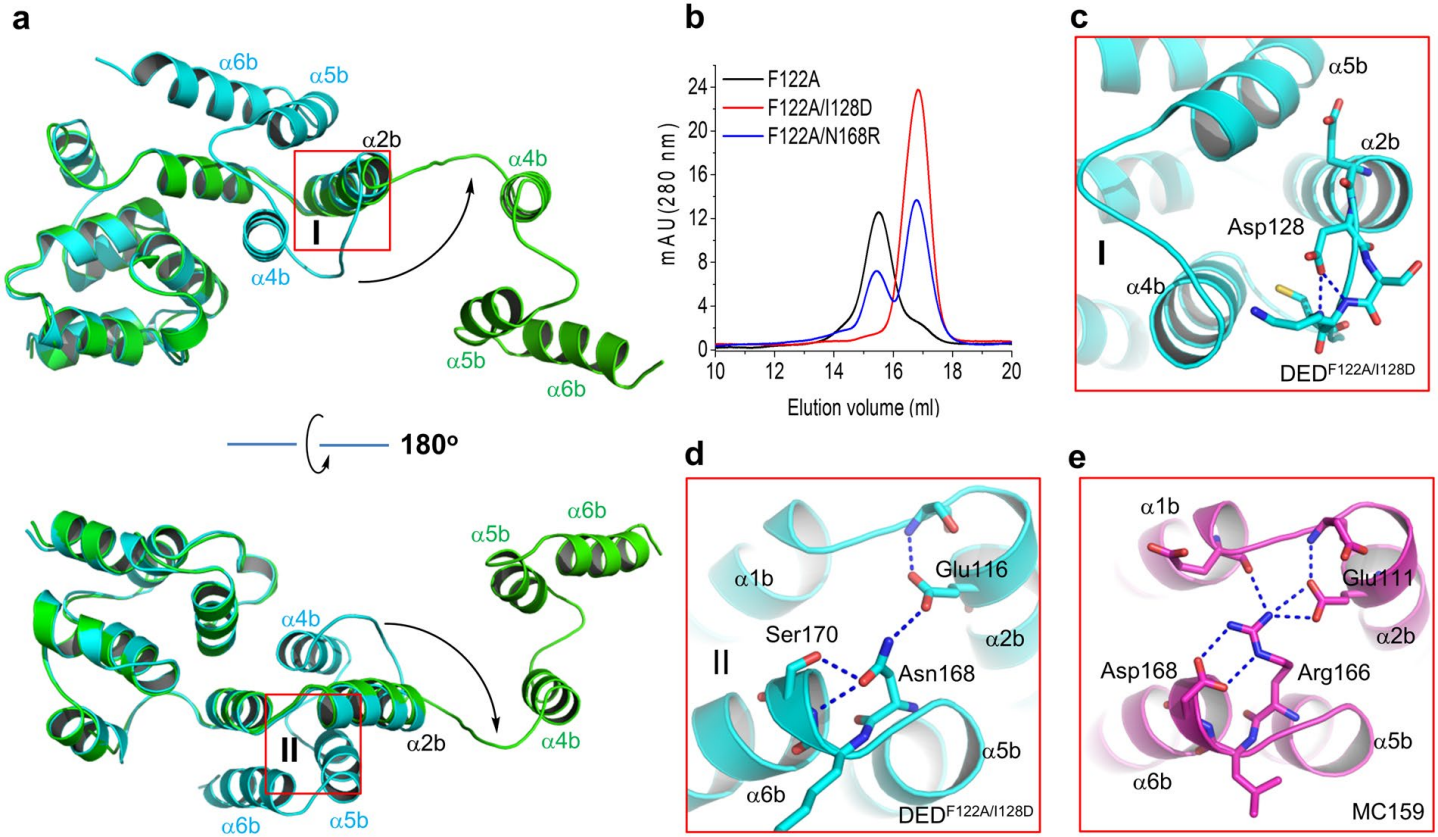
Conformational changes in domain swapping of DED^F122A^. (**a**) Superposition of structures of the monomeric subunit of the domain-swapped dimer of DED^F122A^ (green) and the monomeric DED^F122A/I128D^ (cyan; PDB ID: 4ZBW). Black arrows highlight the flipping of helices α4b-α6b during domain swapping. (**b**) Gel filtration profiles of DED^F122A^, DED^F122A/I128D^, DED^F122A/N168R^. Both I128D and N168R mutations inhibit the dimerization of DED^F122A^. (**c**) Close-up view of hydrogen bonds formed by I128D with the hinge loop in the monomeric DED^F122A/I128D^. Hydrogen bonds are indicated by blue dashed lines in all figures. (**d**) Close-up view of hydrogen bonds formed by the E-NxSL motif in DED^F122A/I128D^. (**e**) Close-up view of hydrogen bonds formed by the E/D-RxDL motif in vFLIP MC159 (PDB ID: 2BBR).

We next compared the structure of the monomer subunit of the domain-swapped DED^F122A^ dimer with that of the monomeric DED^F122A/I128D^. Both structures are essentially identical except for helices α4b-α6b, which rotate as a rigid body around the helix α2b from the closed conformation in the monomeric DED^F122A/I128D^ to the open conformation in the domain-swapped DED^F122A^ dimer (Supplementary Fig. 1d; Fig. 3a). The opening of helices α4b-α6b exposes about 2,500 Å^2^ solvent-accessible surface area, suggesting a high activation energy barrier for the transition between the DED^F122A^ monomer and domain-swapped dimer, as reflected by the observation that both the DED^F122A^ monomer and domain-swapped dimer are highly stable at room temperature without obvious inter-conversion in days (Fig. 1b).

To further validate that the domain-swapped dimeric structures are not the artifact by the crystal packing, we generated a structure-based Q125C mutation on the helix α2b to facilitate the formation of an intermolecular disulfide bond between the two subunits of the domain-swapped dimer (Supplementary Fig. 3a). The SDS-PAGE analysis indicates that DED^F122A/Q125C^ migrates as both dimer and monomer in the absence of the reducing agent β-mercaptoethanol (βME), while runs as the monomer in the presence of βME, suggesting the disulfide bond formation between the two subunits in the absence of reducing agent. In contrast, F122A mutant runs as mainly a monomer in the absence or presence of βME (Supplementary Fig. 3b). We tried to perform the similar SDS-PAGE analysis for the wildtype DEDs (DED^WT^) and the Q125C (DED^Q125C^) mutant. No dimer staining for both WT and Q125C DEDs no matter in the absence or presence of βME, which might be attributed to the low concentration of the proteins and the potential interference of the mixed slyD.

### Point mutations in DED2 disrupt the dimerization of DED^F122A^

The domain swapping in dimeric DED^F122A^ involves the exchange of helices α4b-α6b in DED2 of DED^F122A^. To understand the potential factors underlying the domain swapping, we tried to identify the point mutations disrupting the dimerization of DED^F122A^. In the previous study^15^, Ile128 has been identified as the key residue that confers the insoluble and aggregation-prone property of DEDs of caspase-8. Here I128D mutation do markedly inhibit the dimerization and oligomerization of DED^F122A^ (Fig. 3b). Ile128 is located at the connection between the helix α2b and the hinge loop. The side chain carboxylate group of I128D form two favorable hydrogen bonds with the backbone N-H of the hinge loop, which would restrain the rotation of the hinge loop and inhibit the domain swapping (Fig. 3c). The furthermore sequence alignment and structural analysis reveals that the corresponding residue of Asp128 in DED^F122A/I128D^ is Asn123 in the viral DED homologue vFLIP (Supplementary Fig. 4a). Similar to Asp128 in DED^F122A/I128D^, Asn123 also forms a hydrogen bond with the backbone N-H of the helix α3b in vFLIP (Supplementary Fig. 4b,c), which might account for the monomeric preference of vFLIP^16,17^. Moreover, Arg166 in the conserved E/D-RxDL motif on helices α2b and α6b of vFLIP forms extensive hydrogen bonding interactions between helices α2b and α6b, whereas the corresponding residue Asn168 in DED^F122A^ would disrupts these interactions, and facilitate the opening of helices α4b-α6b and domain swapping of DED^F122A^ (Fig. 3d,e; Supplementary Fig. 4d). As expected, the N168R mutation significantly inhibits the dimerization of DED^F122A^ (Fig. 3b).

### Dimer formation is important for the filament formation of DEDs

**Figure 4:**
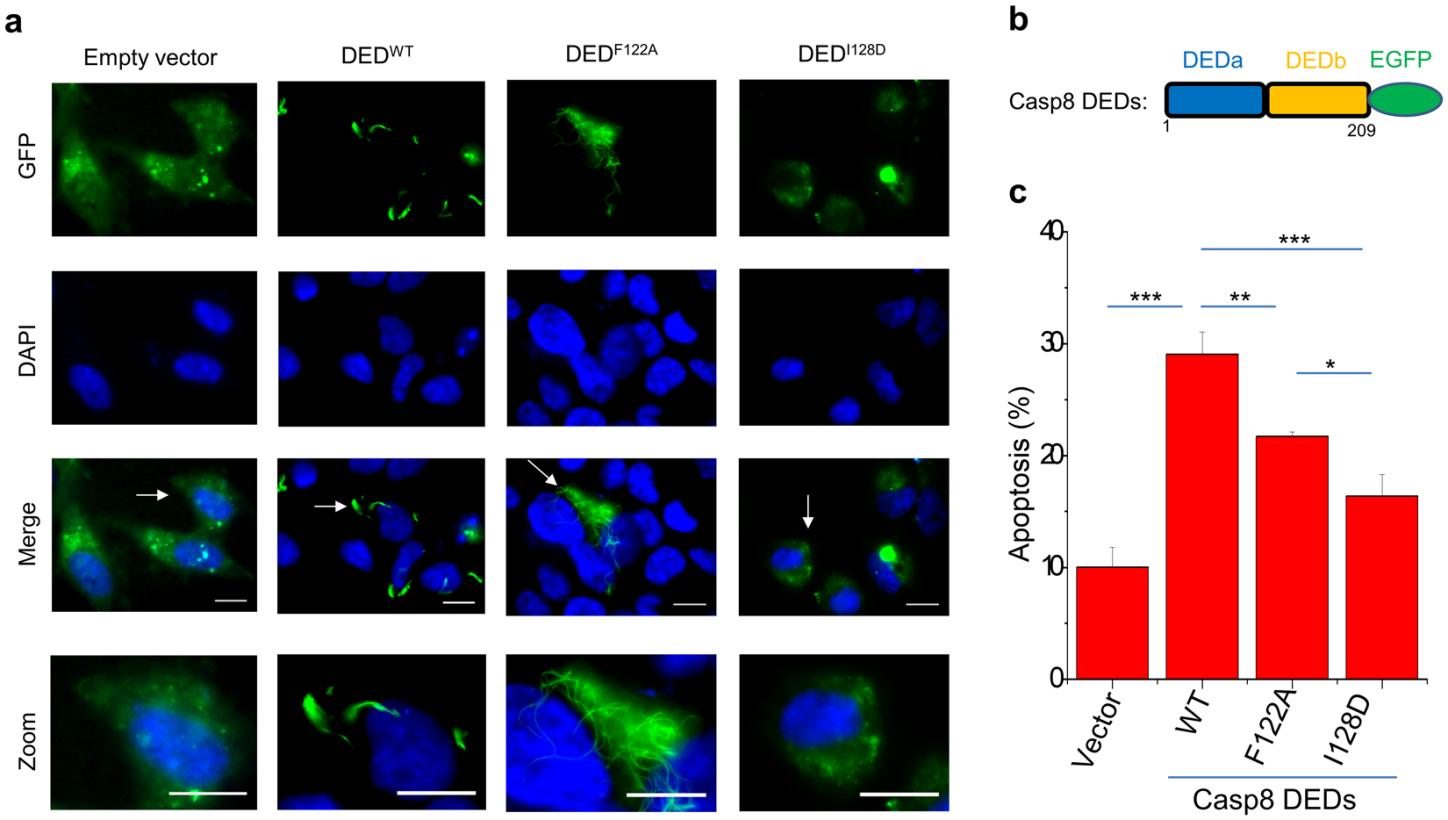
Cellular death effector filament formation. (**a**) HeLa cells were transfected with either empty vector (EGFP) or the caspase-8 DED variants DED^WT^-EGFP or DED^F122A^-EGFP or DED^I128D^-EGFP for 18 hr before fixing and staining with DAPI. Cells were imaged and a representative field for each transfection is shown. Lower panels show enlargement of those areas arrowed in the merge panels. Scale bar, 20 μm. (**b**) Domain schematics of the caspase-8 DED variants fused with EGFP. (**c**) Apoptosis induction of HeLa cells by the caspase-8 DED variants. Apoptosis rates were quantified as the percentage of Annexin V-APC positive versus GFP positive cells. Error bars, s.d. of independent experiments (*n* = 3). *P < 0.05, **P < 0.01, ***P < 0.001 (One-way ANOVA).

The tandem DEDs of caspase-8 have been shown to form novel cytoplasmic death effector filaments (DEFs) upon transient overexpression^19–22^. We thus examined whether the dimerization is important for the DEFs formation (Fig. 4a,b). Compared with the wild-type caspase-8 DEDs, the I128D mutation abrogated the DEFs formation for the tandem DEDs of caspase-8, and markedly reduced the apoptosis of HeLa cells induced by the DEFs (Fig. 4c), highlighting the essential role of the domain-swapped dimerization in the DEFs formation. Intriguingly, DED^F122A^ still formed the distinctive filamentous structure but with a more diffusive pattern (Fig. 4a), which is consistent with the *in vitro* feature of DED^F122A^ that still forms dimer but much less aggregates (Fig. 1a). The DEFs formation and induced apoptosis are less prominent for DED^F122A^ compared with the wild-type DED^WT^, suggesting a potential role of Phe122 in the high-order aggregation of DEDs of caspase-8.

The recent cryo-EM structure has revealed the important role of Phe122 in the formation of the filamentous structure by the tandem DEDs of caspase-8 *in vitro*, in which Phe122 mediates the type I interaction between different DEDs units in the filament (Supplementary Fig. 5a)^18^. This model might also account for the role of Phe122 in the cytoplasmic DEFs formation. On the other hand, this model does not explicitly explain that the I128D mutation abrogates the DEFs formation, since Ile128 is not directly involved in any types of interactions in the filamentous structure (Supplementary Fig. 5b), and no obvious structural change occurs for the tandem DEDs caused by the I128D mutation (Supplementary Fig. 5c). Alternatively, we tried to reconcile the roles of Phe122 and Ile128 in the DEF formation based on the domain-swapped model. Domain swapping has been recognized as an aggregation mechanism for a number of proteins^26,27^. Both dimeric DED^F122A^ exhibit closed-ended domain-swapped structures, which would block the oligomerization of the DEDs and the formation of DEFs. Based on the previous studies^28–30^, it would be reasonable to propose that the closed-ended domain-swapped structures can switch to the open-ended domain-swapped structures and form the final polymerized filaments due to the flexible hinge loop (Supplementary Fig. 6). The I128D mutation disrupts the domain swapping thus in turn blocks the filament formation, and the burial of the hydrophobic residue Phe122 in the wildtype DEDs would more favor the filament formation compared with F122A in this study and F122E in the previous study^18^. Moreover, the DEDs of the full-length caspase-8 could also be integrated into the filaments, leading to the activation of caspase-8 and the downstream apoptosis (Supplementary Fig. 7)^19^.

### Dimer formation of DEDs is essential for the Fas pathway

**Figure 5:**
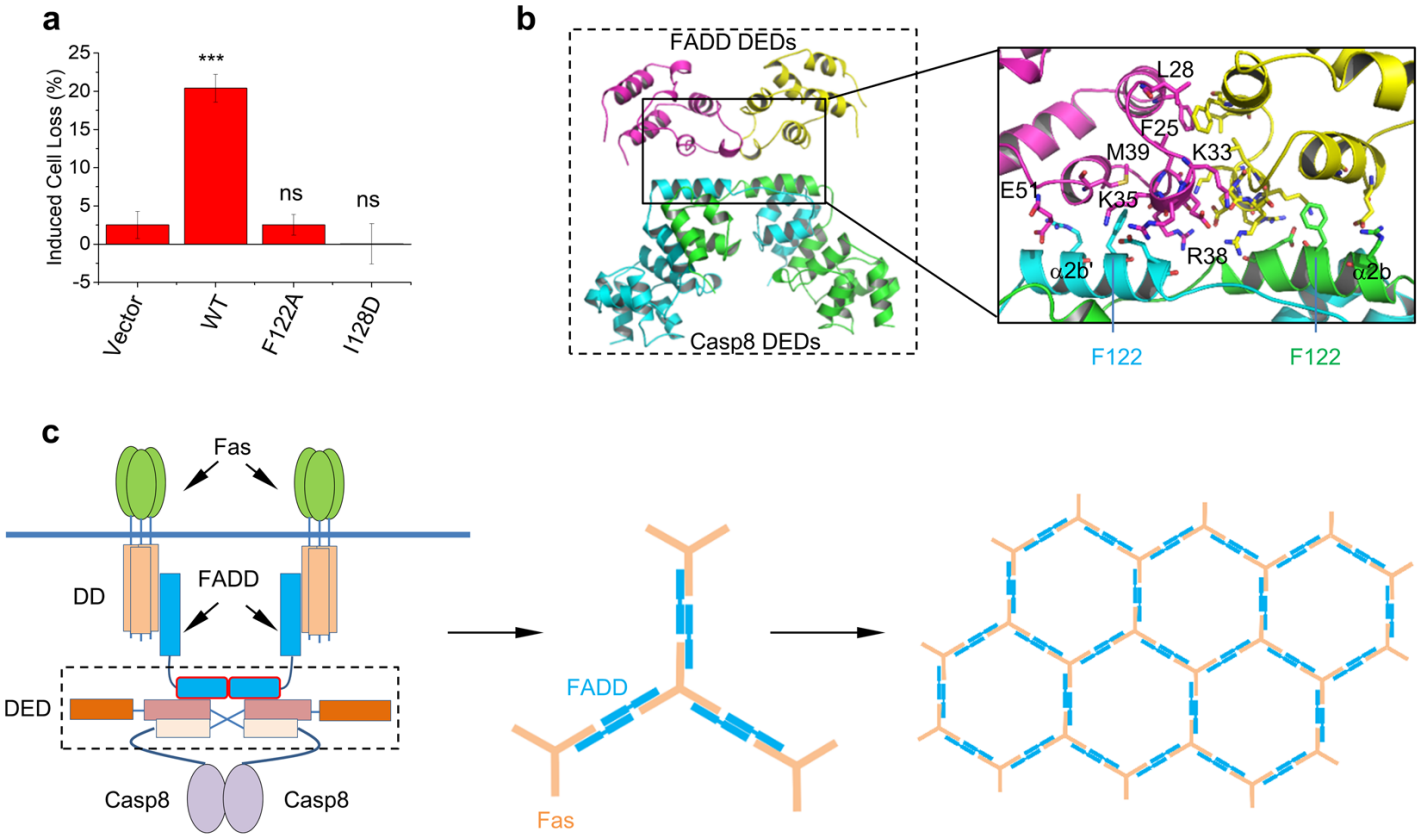
Model of proximity-induced dimerization of caspase-8. (**a**) Apoptosis induction of caspase-8-deficient Jurkat T-cells transfected with the full-length caspase-8 variants by agonistic Fas antibody. The percentage cell loss of GFP^+^ cells that are negative for annexin V was quantitated. Error bars, s.d. of independent experiments (*n* = 3). ns, not significant, ***P < 0.001 (One-way ANOVA). (**b**) Modeled structure of the self-associated dimer of FADD DEDs binding with the domain-swapped dimer of caspase-8 DEDs. Inset, details of the interactions at the dimeric interface of FADD DEDs and the binding interface between DEDs of FADDs and caspase-8. Key residues involved in interactions are highlighted by stick representation. (**c**) Fas receptor clustering array bridged by the self-associated FADDs (middle and right). Fas and FADD are colored in wheat and marine blue, respectively. Minimal DISC model composed of dimeric Fas trimers, dimeric FADDs and caspase-8s is shown in the left panel. The prodomains of caspase-8 exist in a domain-swapped dimer.

To further assess the physiological relevance of the domain-swapped dimer of DEDs, we determined the ability of anti-Fas agonist antibody to induce apoptosis of caspase-8-deficient Jurkat cells transfected with empty vector (EV) and wild-type (WT), F122A and I128D mutant procaspase 8 (Fig. 5a). Transfection of wild-type caspase-8, but neither F122A nor I128D mutants, rescued the apoptotic response of caspase-8-deficient Jurkat cells towards anti-Fas agonist antibody. The defect of I128D mutant highlights the crucial role of the domain-swapped dimerization of DEDs of caspase-8 in the extrinsic apoptotic pathway. On the other hand, though F122A mutant has the capability to form dimer in solution and in the crystal, and even to form intracellular filaments, it was severely impaired in rescuing the cellular apoptosis of caspase-8-deficient Jurkat cells induced by anti-Fas agonist antibody, suggesting a distinct role of Phe122 rather than promoting dimer and filament formation in the extrinsic apoptotic pathway.

Phe122 of caspase-8 has been shown to be important for interaction with FADD DED^15,18^ and the recruitment of caspase-8 to the DISC^31^, but it is unclear how Phe122 mediates the interaction between caspase-8 and FADD via their DEDs at the DISC, and how the dimerization of the DEDs of caspase-8 involves in this interaction. The previous study^32^ has indicated that FADD self-association via the DED is required for stable interaction of FADD with Fas and with caspase-8. The replacement of the DED of FADD by a dimeric leucine-zipper coiled-coil maintains the capability of the death domain (DD) of FADD to bind with the Fas DD, suggesting that the dimerized DEDs of FADD might be the functional unit for the interactions between the DDs of FADD and Fas. In line with this hypothesis, the dimerized DEDs of FADD might serve as the docking site of the domain-swapped dimer of the DEDs of caspase-8 and facilitate the recruitment of caspase-8 to the DISC.

To understand the potential binding mode between FADD and caspase-8, we used molecular modeling to generate a complex model between the DEDs of FADD and caspase-8. Given a small cluster including Phe25, Leu28 and Lys33 critical for the self-assembly of FADD, we first built up the dimeric structures of the DEDs of FADD with this cluster as the interfacial residues. Using Phe122 of the opened domain-swapped dimeric DEDs of caspase-8 as the anchoring points, a potential complex model was generated for the dimeric DEDs of FADD and caspase-8 (Fig. 5b). The solvent exposed Phe122 of caspase-8 fits well into the hydrophobic groove lined by the residues on the helices α3 and α4 of FADD, which is consistent with the proposed interactions between FADD and caspase-8. The dimerization of the DEDs of both FADD and caspase-8 strengthens the interaction between FADD and caspase-8, and vice versa. This model allows the integration of the full-length caspase-8 and provides insight into the proximity-induced dimerization mechanism of caspase-8 activation within the DISC (Supplementary Fig. 8).

## Discussion

DISC formation and subsequent caspase-8 activation are critical initial events in the extrinsic apoptotic pathway^7^. Extensive previous studies have provided significant insights into understanding this process, but the underlying mechanisms still need further investigation and continuing refinement^18,21,22,32–34^. In this study, we found that the tandem DEDs of caspase-8 form a novel domain-swapped dimer, in which the domain-swapping involves the exchange of the C-terminal helices α4b-α6b between the two subunits. Employing functional studies we demonstrated that dimerization of the DEDs is essential for activation of caspase-8. Furthermore, though the conserved residue Phe122 is not necessary for the dimerization, it is essential for the activation of caspase-8 and the downstream apoptosis cascade.

Our work supports the proximity-induced activation model of caspase-8^5–7^. The domain-swapped dimerization of the DEDs brings the protease domains into proximity and facilitates the dimerization and activation of caspase-8, which closely resembles that occurs in the activation of the chimeric FKBP-caspase-8 induced by the dimerizing agent FK1012^9^. Moreover, upon engaging with the DED of FADD, the domain-swapped dimer of the DEDs of caspase-8 are also easily integrated into the clustered Fas/FADD complexes in which trimeric Fas assemble into arrays bridged by dimeric FADD (Fig. 5c)^32,35^. This model explicitly explains the observation that dimerized FasL trimers have about 1000-fold enhancement of the signaling efficiency compared with the non-crosslinked FasL trimer^10^. This model is also consistent with the observation that the membrane-bound CD95 ligand rather than the soluble one is critical for apoptosis^36^. We noted that it is currently unclear whether the domain-swapped dimerization of the DEDs of caspase-8 occurs before or upon binding with FADD in the DISC, and it is also unclear how the cytosolic monomeric DEDs switches to the domain-swapped dimer upon the upstream death signal in physiological condition.

It is not uncommon to observe the domain swapping of functional proteins in the death domain superfamily. For example, the N-terminal caspase-recruitment domain (CARD) of Nod1 forms a domain-swapped homodimer by swapping the H6 helices at the carboxy termini^37–39^. Moreover, the N-terminal pyrin domain (PYD) of NLRP14 also forms a homodimer by a rearrangement of the α5/6 stem-helix of the C-termini^40^. In addition, domain swapping has been well described for the dimerization/oligomerization of both the pro-apoptotic proteins Bax/Bak^41,42^ and the pro-survival protein BCL-XL^43^ in the intrinsic apoptosis pathway, in which the transition from the monomer to the domain-swapped dimer is facilitated by the detergents or membrane lipid. We speculated that the domain-swapped dimerization of the DEDs of caspase-8 might also be facilitated by the membrane lipid when recruited to the membranous DISC, or possibly assisted by yet identified chaperones^44–46^.

DEDs can form death effector filaments (DEFs) intracellularly or *in vitro* at the high concentration of DEDs^18–22^. The recent cryo-EM structure of caspase-8 tandem DED filaments has revealed that the DEFs formation likely occurs through repeated self-assembly by type I, II, and III interactions of the DEDs, in which both DED1 and DED2 are critical for the self-assembly^18^. This structure does not provide a clear understanding for the previous study that only DED2 but not DED1 still forms fine cytoplasmic filaments^19^, while this discrepancy can be explained by the domain-swapped filament model in which only DED2 is involved in oligomerization (Supplementary Fig. 6c). On the other hand, the filamentous structure generally forms under high concentrations of DEDs with the length of about 100 nm or even longer^18–22^, raising a question about its physiological relevance. Given a limited number of caspase-8 in cell under physiological condition, the probability of such long filamentous structure for caspase-8 is very low during the apoptotic pathway triggered by the death ligand. Consistent with this analysis, the DEDs of caspase-8 forms spot-like rather than filamentous structure under a low overexpression level upon FasL stimulation^22^, more likely reflecting the arrayed FADD in signaling protein oligomerization transduction structures (SPOTS)^32,35,47^. This observation can be easily explained by our model in which the domain-swapped dimer of the DEDs of caspase-8 bind with the dimeric DEDs of FADD in the membranous DISC, the fluorescent signal of caspase-8 therefore reflects the array of FADD. In contrast, upon a high level of overexpression of the DEDs of caspase-8, the cytoplasmic DEFs were detected even without FasL stimulation^18–22^.

In summary, we reported the crystal structures of a domain-swapped dimer of the DEDs of caspase-8. Together with functional analysis, we demonstrated that the domain-swapped dimerization is critical to the activation of caspase-8. Furthermore, we generated a potential complex model for the homotypic interactions between the DEDs of FADD and caspase-8, which would shed new light on the DISC in the extrinsic apoptotic pathway. Meanwhile, the domain-swapped dimerization represents the fourth homotypic interaction type, together with other three known interaction types, for understanding the functions of proteins in the death-fold superfamily.

## Methods

### Reagents and cell lines

The monoclonal agonist antibody against CD95 (Anti-Fas) was purchased from Millipore (Bedford, MA). Caspase-8 deficient Jurkat cells were kind gift from Dr. Junying Yuan at Harvard University. HeLa cells were obtained from Cellbank (Shanghai, China). Jurkat cells were cultured in RPMI1640 and HeLa cells were cultured in DMEM (Corning, Manassas, VA), supplemented with 10% fetal bovine serum (PAN-Biotech, Aidenbach, Germany). All the other biochemical reagents were from Sigma Aldrich.

### Protein expression and purification

The DNA encoding the death effector domain (DED) of human caspase-8 (residues 1–188, Uniprot accession number Q14790-1) were subcloned into a pET-21b vector with a C-terminal His6 tag or subcloned into a pET-28a vector with an N-terminal His-SUMO tag by a standard PCR method. The relevant mutants of Caspase-8 DED (residues1-188) were generated by Fast Mutagenesis System (TransGen Biotech, China). All the Caspase-8 DED constructs mentioned above were transformed into Rosetta (DE3) pLysS cells (Novagen). All of the recombinant protein expression were induced with 0.1 mM IPTG at 20 °C overnight. His-tagged proteins were purified with Qiagen Ni-NTA agarose according to the manufacturer’s instructions. After the first step purification, His-SUMO tag was removed by ULP-1 enzyme, and the proteins were re-chromatographed on a Ni-NTA column. The His-SUMO-tag-cleaved protein was further purified by gel-filtration chromatography (Superdex 200 10/300 GL, GE Healthcare).

### Crystallization, data collection and structure determination

The dimeric peak of DED^F122A^ was collected and concentrated to 6–8 mg/ml in buffer A (20 mM Tris, pH 8.0, 1 mM DTT and 150 mM sodium chloride) for crystallization. Using hanging drop vapor diffusion method, crystals of DED^F122A^ in two crystal forms grew in a few days in condition A (50 mM sodium chloride, 100 mM Tris pH 8.5, 22.5% PEG3350, at 20 °C, for the crystal form A with short cell dimension and the crystal structure in the open conformation) and condition B (100 mM sodium chloride, 100 mM Tris, pH 8.5, 21% PEG3350, 10 mM Sarcosine, at 20 °C, for the crystal form B with long cell dimension and the crystal structure in the closed conformation), respectively. For cryo-freezing of the crystals, 20% glycerol in mother liquor was used as Cryo-protectant by multi-steps transferring. The crystals were flash frozen in liquid nitrogen for a further X-ray diffraction data collection.

The diffraction data for the crystal form A were collected in homesource Rigaku micromax-002+. The diffraction data for the crystal form B were collected at beamline BL17U of Shanghai Synchrotron Radiation facility. Data were integrated, scaled and processed by HKL2000^48^. The detailed statistics are shown in Extended Data Table 1.

The crystal structure was determined by molecular replacement carried out by Phaser-PHENIX program suite^49,50^, the monomer of DED^F122A/I128D^ (PDB accession code 4ZBW^15^) was used as the initial search model. The final models were manually built in Coot^51^ and refined by Refmac^52,53^ in CCP4. The refinement statistics were listed in Supplementary Table 1. All crystallographic figures were drawn in PyMOL^54^.

### Immunofluorescence assay

HeLa cells were cultured on coverslips in 12 well plates for 24 hr before transfected with 1.4 μg/well of vectors using Lipofectamine® 2000 Reagent (Invitrogen) according to the manufacturer’s instructions. 18 hr later, cells were fixed in Formalin solution for 30 min at room temperature and the nuclei was stained with DAPI for 20 min. Cells were washed in TBS buffer (20 mM Tris, 200 mM NaCl, pH = 7.6) 3 times. Images were taken with AxioImager A1 microscope and AxioCam digital camera (Zeiss, Oberkochen, Germany).

### Apoptosis assays

For cell-based apoptosis assay, the wild type (WT) and F122A, I128D mutants of full length Caspase-8 (residues 1–479) and DEDs (residues1–209) were subcloned into pEGFP-N1 vector. HeLa cells (3 × 10^5^) were transfected with plasmids (4 μg) as indicated in figure legends using TransIn EL Transfection Reagent (TransGen Biotech, China). For Jurkat cells, 2 μg plasmids were electroporated into 1 × 10^6^ cells with Amaxa SE Cell Line 4D-Nucleofector X Kit (Lonza Group Ltd, Basel, Switzerland). 18 hr post transfection/electroporation, cells were further stimulated with anti-Fas (1 μg/ml) for 20 hr before harvest. The cell sample were then analyzed for apoptosis via Annexin V staining (eBioscience) using FACSCalibur flow cytometer (Becton Dickinson, NJ, USA). Apoptosis rates were quantified as the percentage of Annexin V-APC positive versus GFP positive cells.

## Electronic supplementary material

Supplementary information

## Data Availability

The atomic coordinates and structure factors have been deposited at the Protein Data Bank under accession numbers 5H31 and 5H33. The data that support the findings of this study are available within the article and its Supplementary Information files, or available from the corresponding author on request.
