# Supplementary material for: Structural basis for dimerization of the death effector domain of the F122A mutant of Caspase-8
Source: Sci Rep. 2018 Nov 13;8:16723. doi: 10.1038/s41598-018-35153-5 (PMC6233201; doi:10.1038/s41598-018-35153-5)
Supplement: Supplementary file 1 — Supplementary information [file 41598_2018_35153_MOESM1_ESM.pdf]

Structural basis for dimerization of the death effector domain of the F122A mutant of Caspase-8

Chen Shen[1], Jianwen Pei[1], Xiaomin Guo[1], Lu Zhou[2], Qinkai Li[1], & Junmin Quan[1*]
[1] Laboratory of Chemical Genomics, School of Chemical Biology and Biotechnology, Peking University Shenzhen Graduate School, Shenzhen 518055, China
[2] School of Pharmacy, Fudan University, Shanghai 201203, China
Chen Shen, Jianwen Pei and Xiaomin Guo contributed equally.
[*] Correspondence and requests for materials should be addressed to J. Q (email: quanjm@szpku.edu.cn)

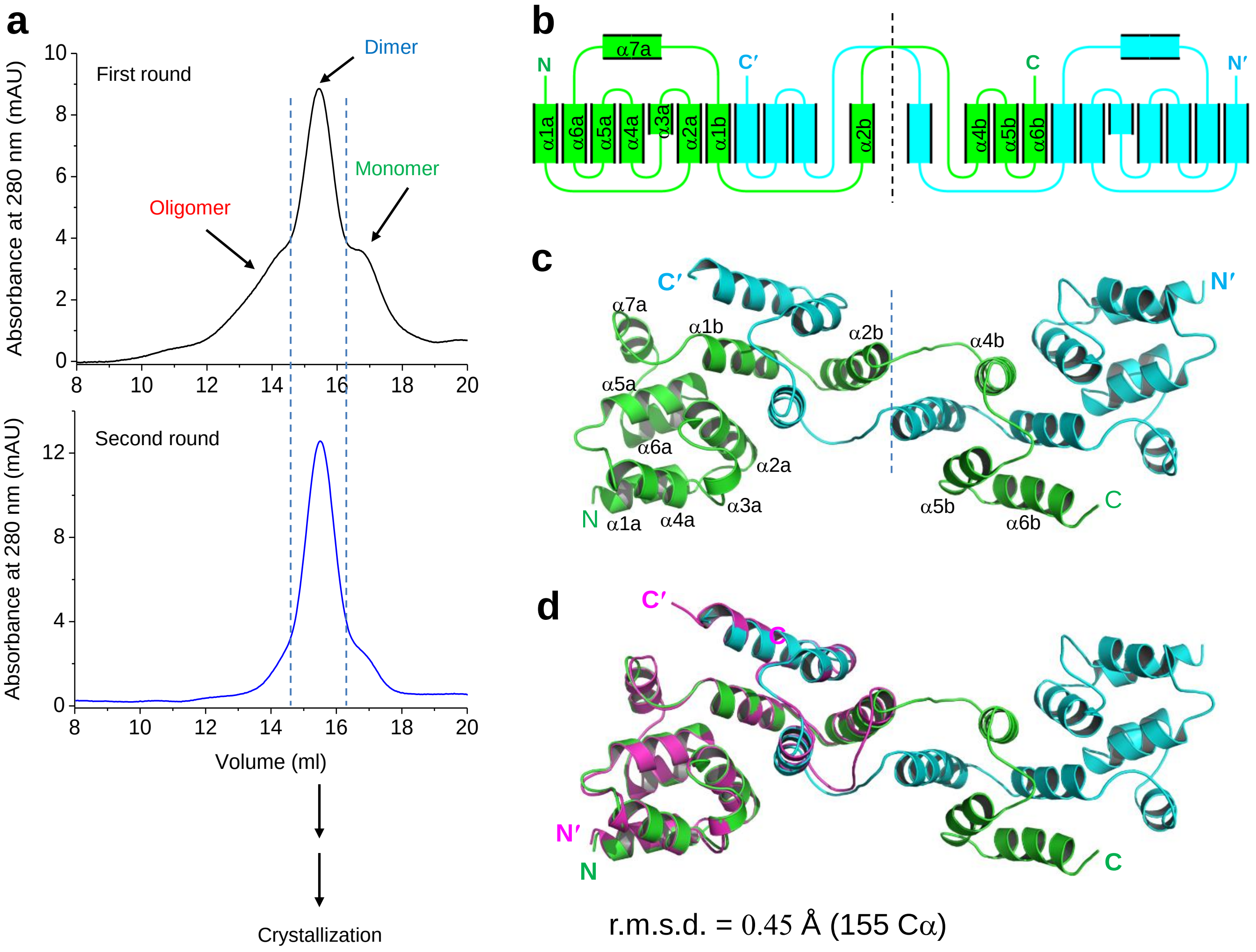

Supplementary Figure 1. DED^{F122A} dimerization. (**a**) Gel filtration profile of DED^{F122A} in successive purifications. Peaks corresponding to monomer, dimer and oligomer are highlighted in the first round of purification. (**b**) Topology diagrams of DED^{F122A} domain-swapped dimer. Helices are represented by cylinders and numbered sequentially. The two subunits are colored in green and cyan, respectively. (**c**) Overall structure of the domain-swapped dimer of DED^{F122A} in cartoon representation, illustrating domain swapping of helices α4b-α6b across the dotted line. (**d**) Structural superimposition of the monomeric DED$^{F122A/I128D}$ (PDB ID: 4ZBW) and the domain-swapped dimeric DED^{F122A} in the close conformation (PDB ID: 5H31).

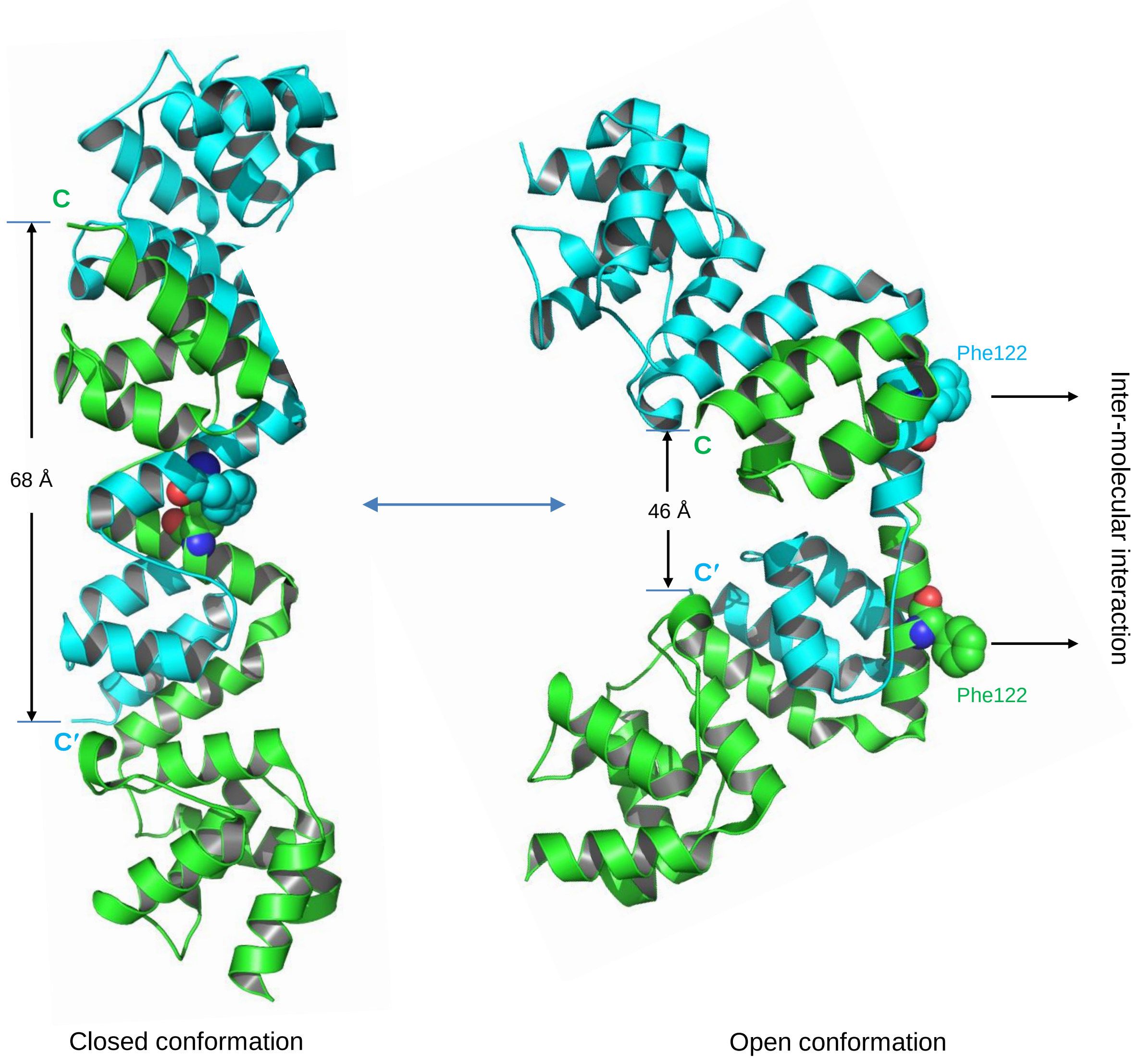

Supplementary Figure 2. Closed and open conformations of the dimeric DEDs. Cartoon representation of domain-swapped dimeric DEDs in the closed (PDB ID: 5H31, left) and the open (PDB ID: 5H33, right) conformations. Phe122 was modeled from F122A and highlighted by sphere representation. The distances between the C-terminus of the two subunit in two different conformations are indicated. The open conformation of the dimeric DEDs exposes the hydrophobic residues Phe122 for inter-molecular interaction, and brings the C-terminal protease domains closer for activation.

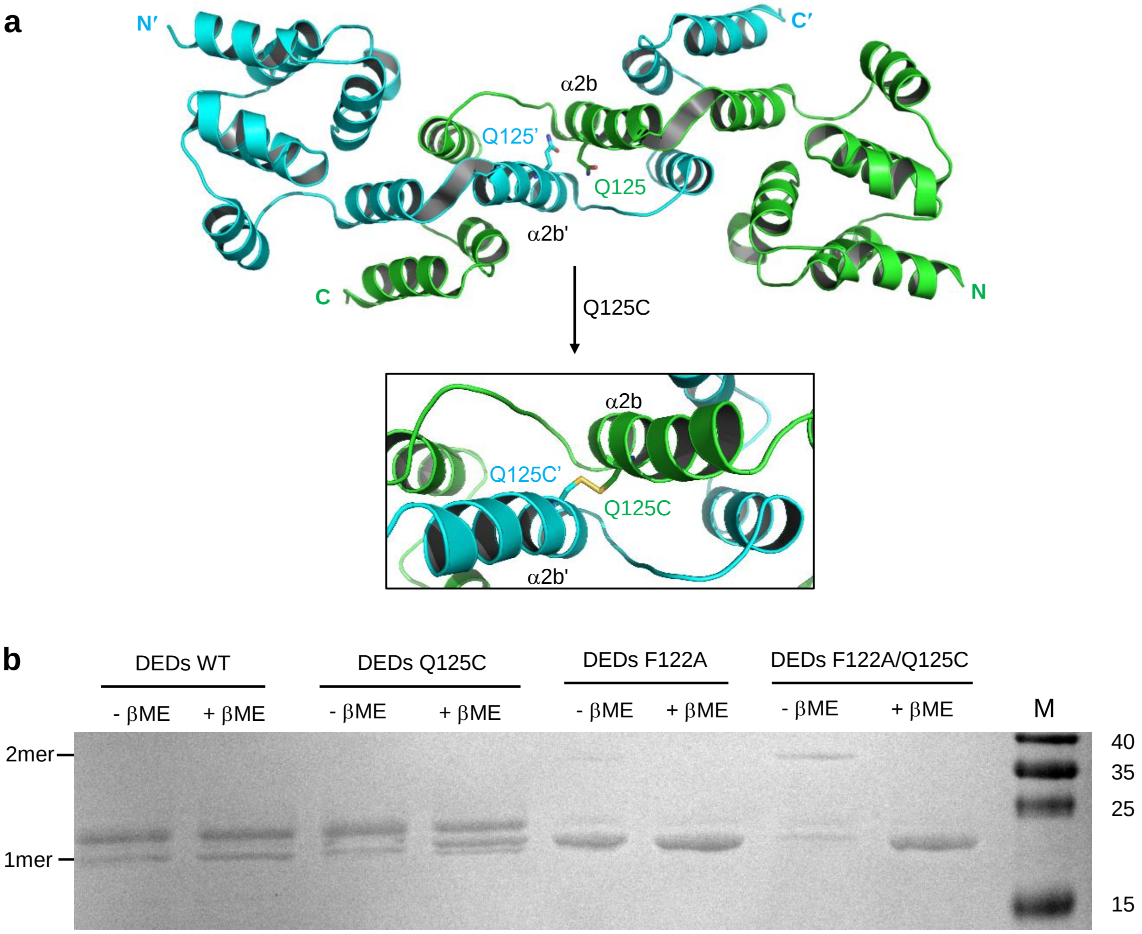

Supplementary Figure 3. Disulfide bond formation of the dimeric DEDs. (**a**) Cartoon structure of the domain-swapped dimer illustrating Q125C mutant to form intermolecular disulfide bond. (**b**) The Coomassie stained SDS gel for WT and mutants of DEDs in the absence and in the presence of beta mercaptoethanol (βME).

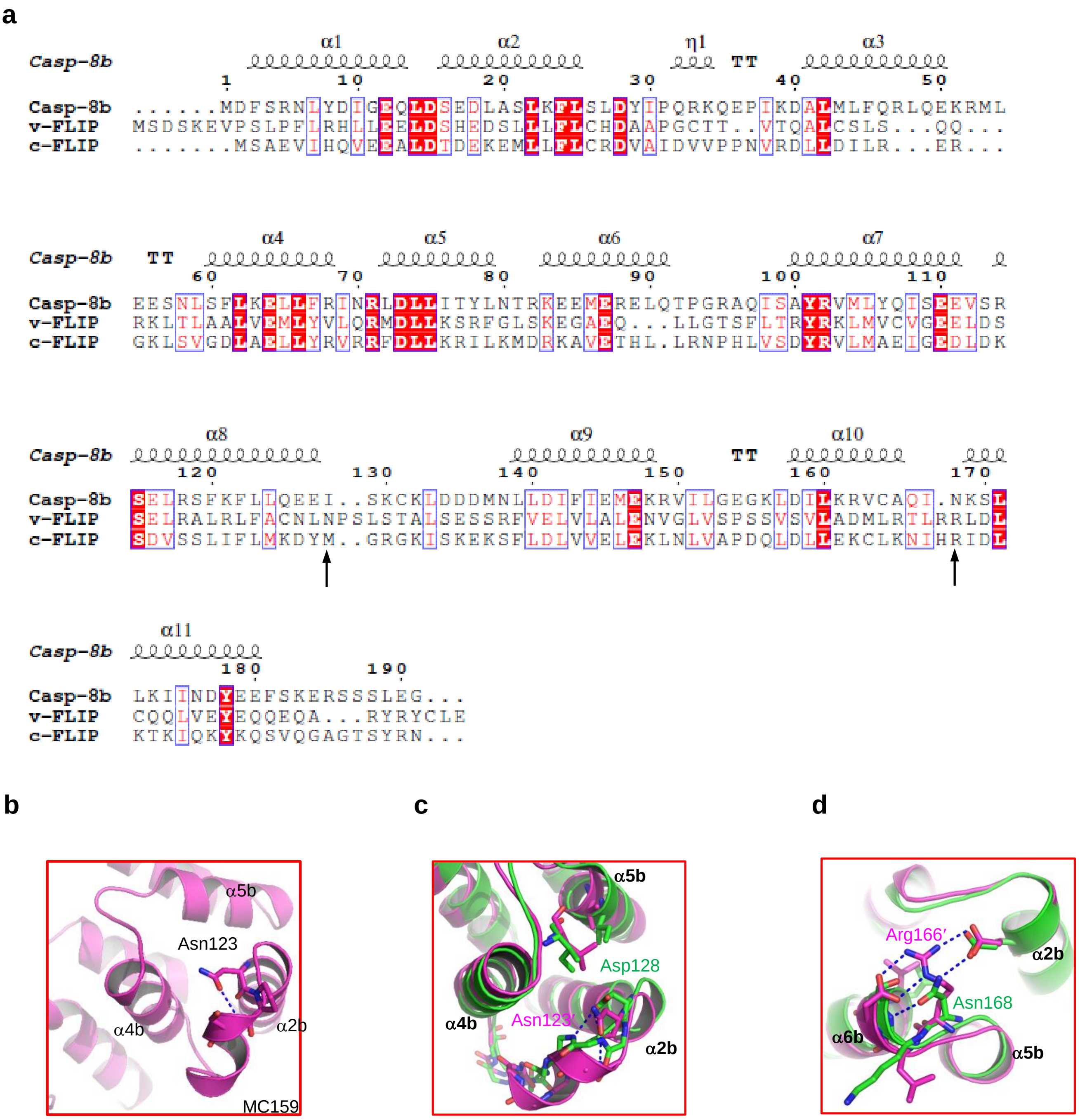

Supplementary Figure 4. Structural comparison between the DEDs of caspase-8 and vFLIP. (**a**) Sequence alignment of the death-effector domains of caspase-8, vFLIP and cFLIP. Black arrows indicate the key residues that affect the domain swapping of the DEDs. (**b**) Close-up view of hydrogen bonds formed by N123 with the hinge loop in vFLIP MC159 (PDB code 2BBR) . **(c)** Structural comparison of the DEDs of caspase-8 (green) (PDB ID: 4ZBW) and vFLIP MC159 (magenta) (PDB ID: 2BBR). I128D in DEDF$^{122A/I128D}$ and Asn123 in vFLIP was highlighted. (**d**) Structural comparison at the conserved E/D-RxDL region. Asn168 in DED^{F122A} and Arg166 in vFLIP was highlighted. Hydrogen bonds were indicated by blue dashed line.

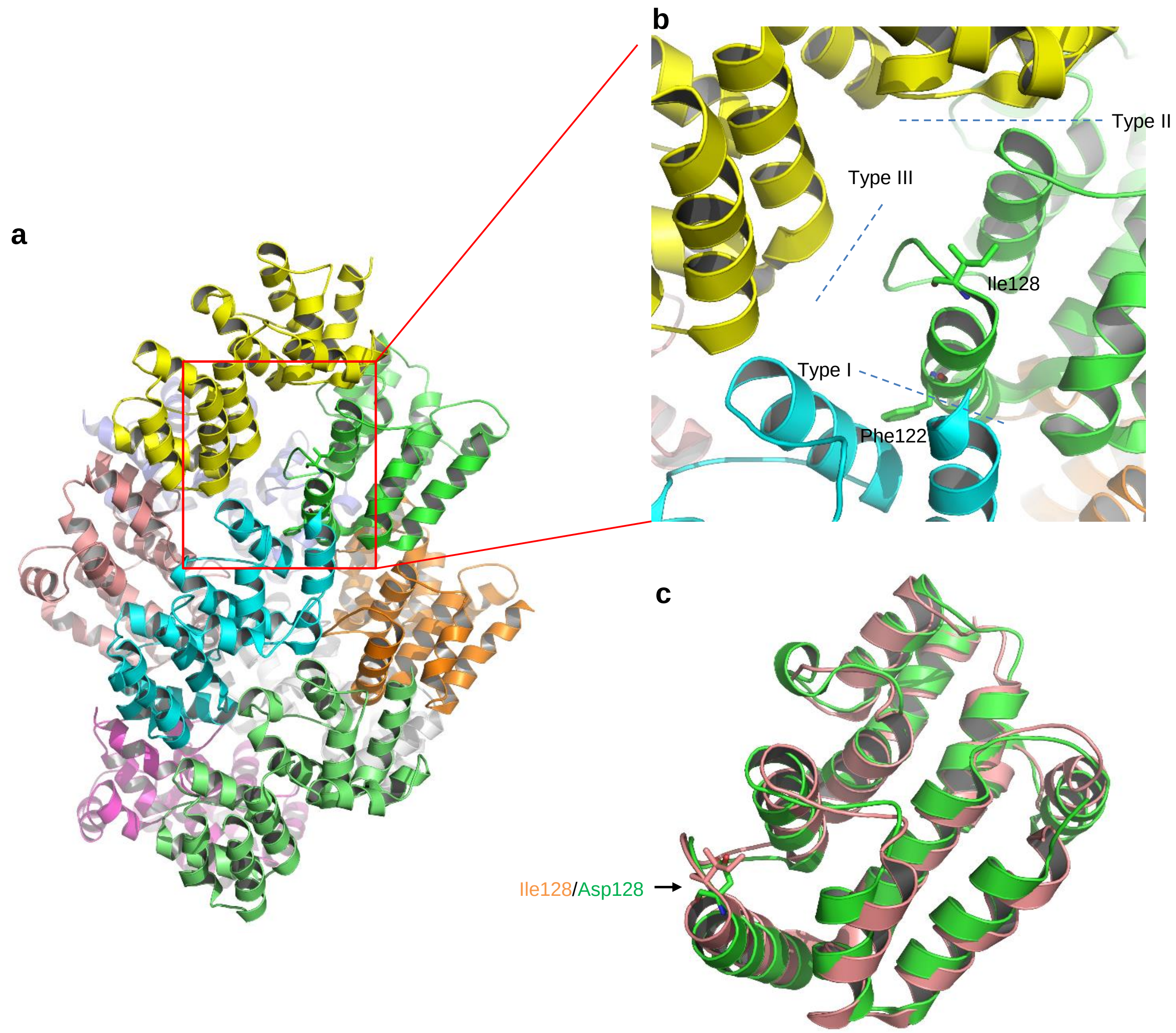

Supplementary Figure 5. Structures of the filamentous DEDs of caspase-8. (**a)** Cartoon representation of the filamentous DEDs of caspase-8 (PDB ID: 5L08), subunits are indicated by different colors. (**b)** Close-up view of the three interaction types in the helical filament. The interfaces are indicated by dash lines. Ile128 and Phe122 are represented by sticks. Phe122 is located at the type I interface, while Ile128 is located outside of the interfaces of the three interaction types. **(c)** Structural superimposition of the monomeric DED$^{F122A/I128D}$ (PDB ID: 4ZBW) and one of the subunits of the filamentous DEDs of caspase-8 (PDB ID: 5L08).

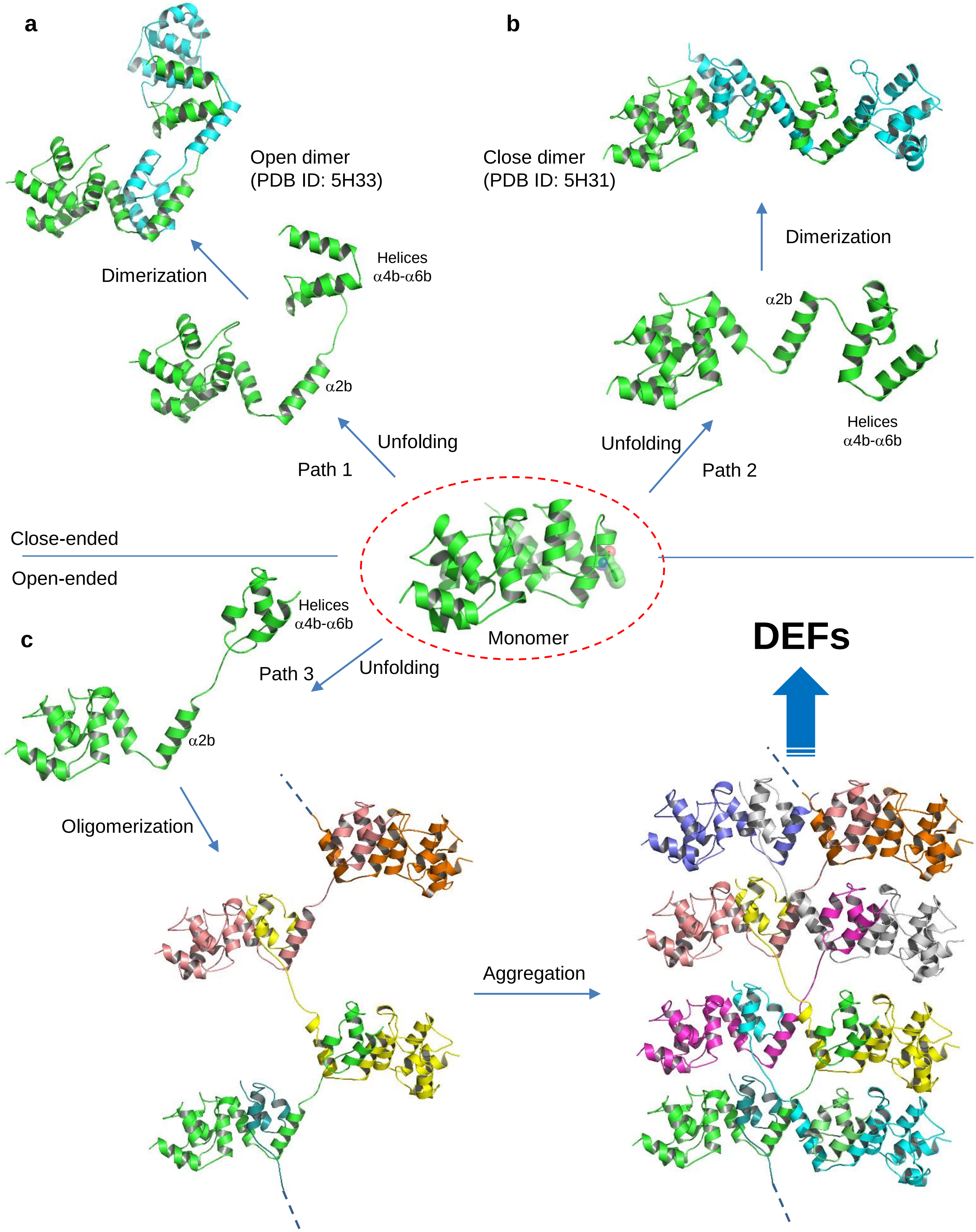

Supplementary Figure 6. Schematic representation of polymerization of the DEDs of caspase-8. (**a)** Close-ended domain swapping to form the open-conformational dimers of the DEDs from the monomeric DEDs. **(b)** Close-ended domain swapping to form the closed-conformational dimers of the DEDs from the monomeric DEDs. (**c)** Open-ended domain swapping to form the oligomers and filaments (DEFs) of the DEDs from the monomeric DEDs. The end products were determined by the orientations of the flexible hinge loop, the unfolding intermediate might be stabilized by molecular chaperones or other unknown cellular factors.

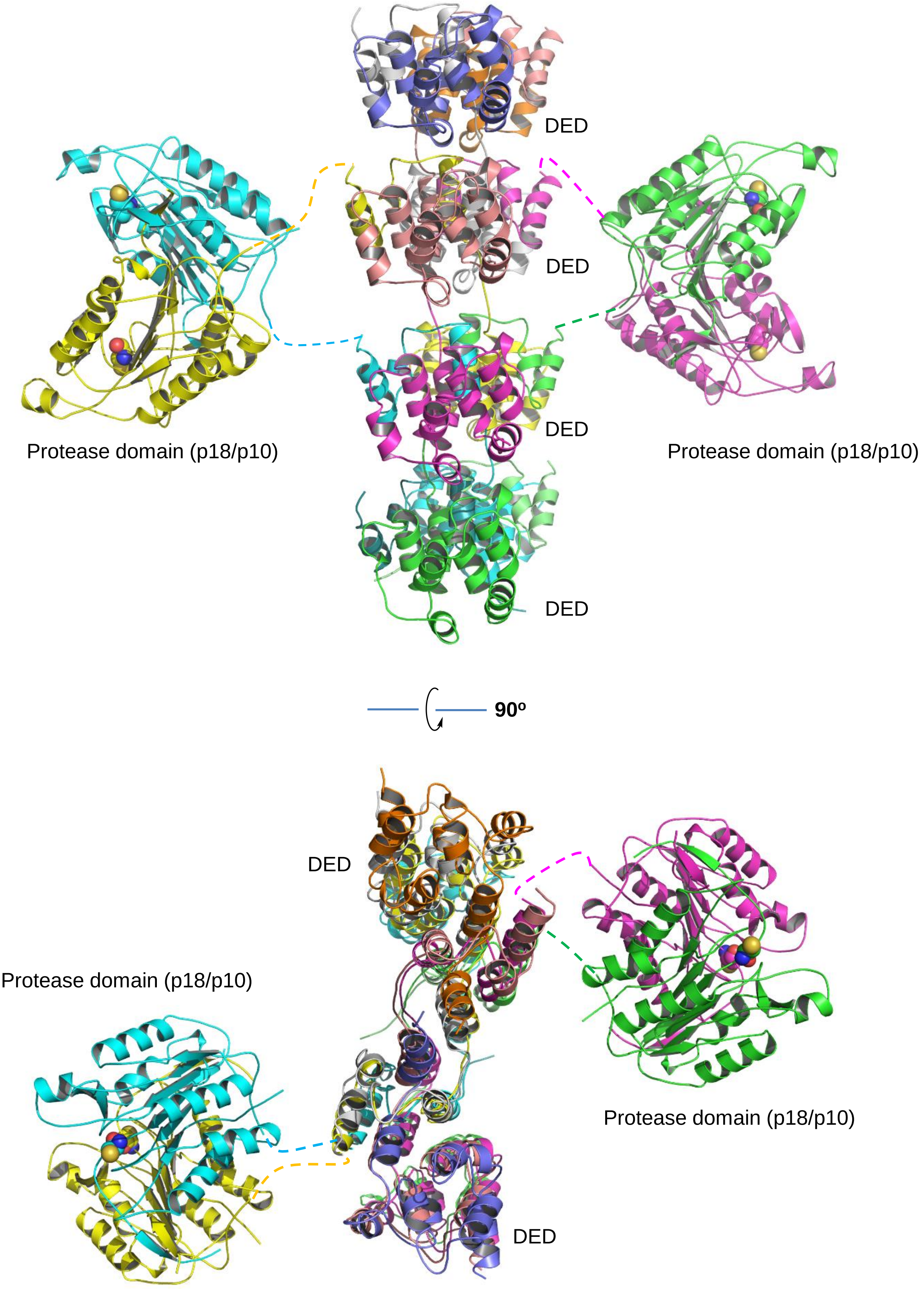

Supplementary Figure 7. Modeled structure of the DEFs of caspase-8 with recruited full-length caspase-8. Side and top views of the modeled structure of DEFs formed by open-ended domain swapping of the DEDs of caspase-8. The recruited full-length caspase-8s from the successive units facilitate the protease domains to form the dimer for activation[5]. The linkers connected the DEDs and the protease domains were indicated by dash lines. The catalytic residue Cys360 in the protease domain was shown by sphere representation. The protease domains were modeled from the crystal structure (PDB ID: 1QTN).

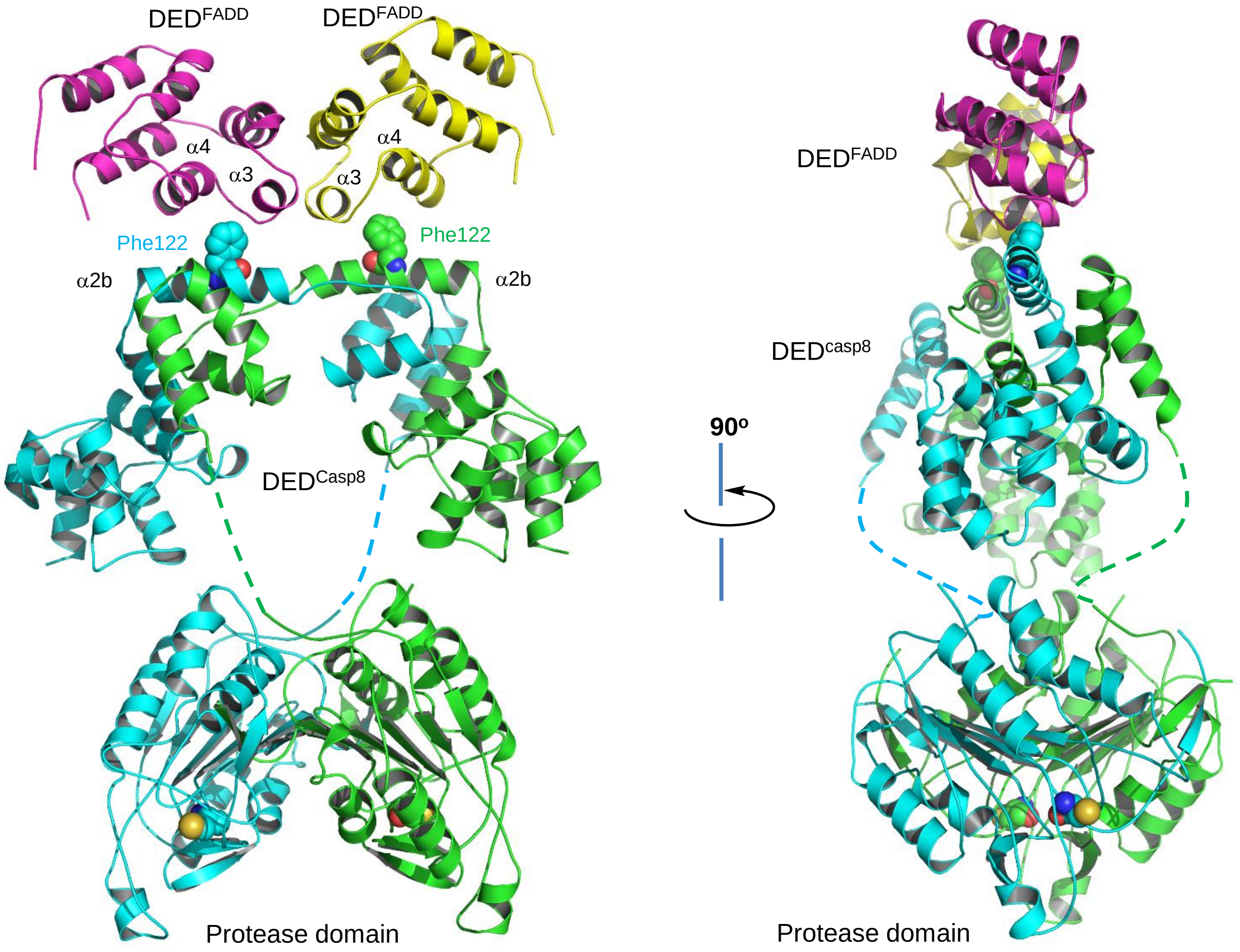

Supplementary Figure 8. Modeled structure of the dimeric full-length caspase-8 binding with the dimeric DEDs of FADD. The binding was mainly mediated by the interactions between helices α2b of the domain-swapped dimer of the DEDs of caspase-8 and helices α3 and α4 of the dimeric DEDs of FADD. The docking Phe122 of the DEDs of caspase-8 was indicated by sphere representation. The linkers connected the DEDs and the protease domains were indicated by dash lines. The domain-swapping dimerization of the DEDs of caspase-8 facilitates the protease domains to form the dimer for activation. The catalytic residue Cys360 in the protease domain was shown by sphere representation.

Full-length gels for Figure 1.

DEDs F122A

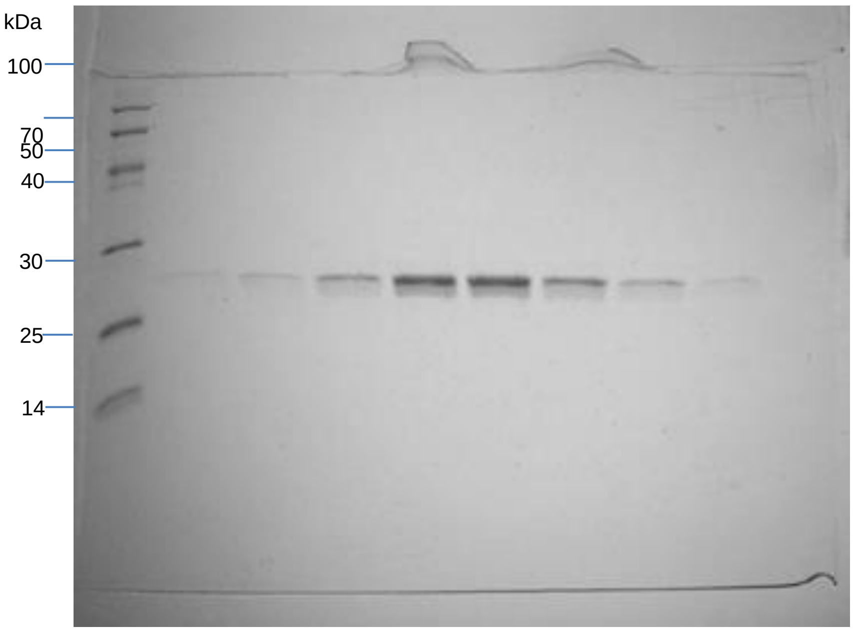

DEDs WT

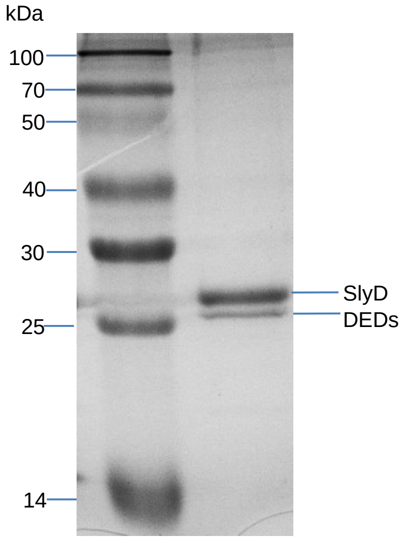

Full-length gel for Supplementary Figure 3.

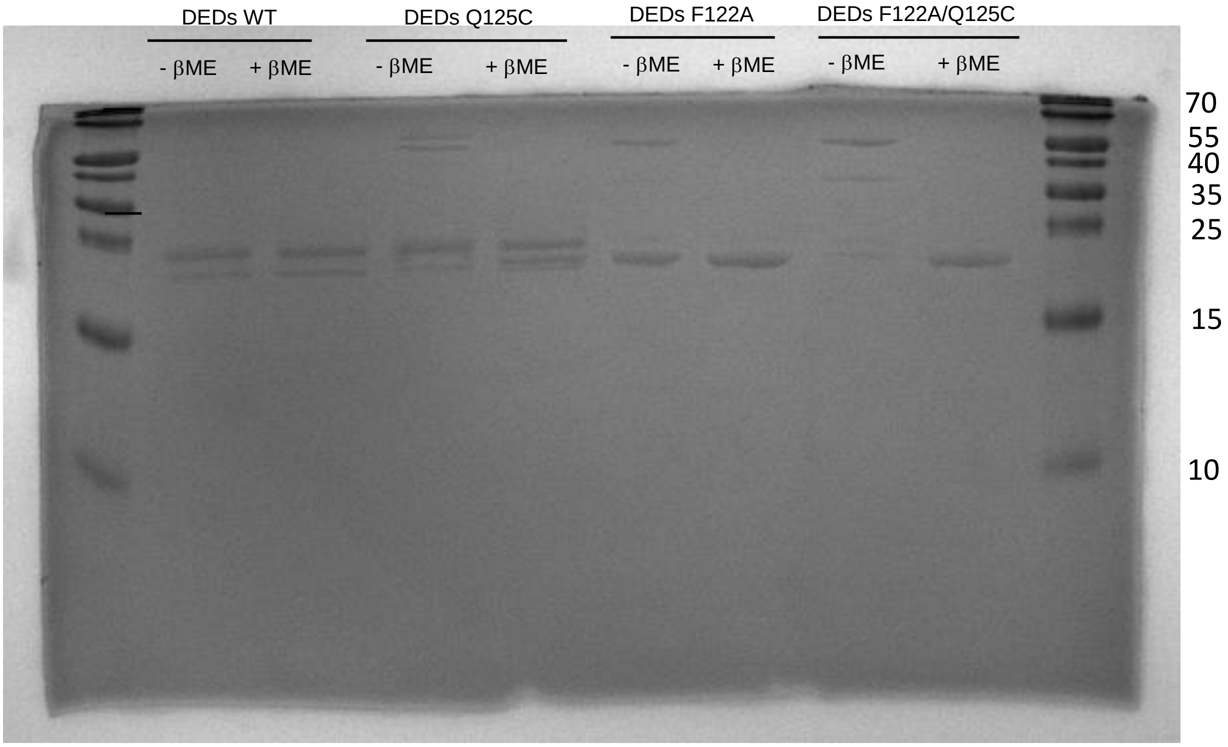

Supplementary Figure 9. Full-length gels
